# A long-term retrospective analysis of oncologic and fertility outcomes in cervical cancer patients undergoing radical trachelectomy

**DOI:** 10.3389/fonc.2025.1591923

**Published:** 2025-09-11

**Authors:** Yu Liu, Xuyin Zhang, Weijuan Xin, Yan Ding, Yunqiang Zhang, Ning Zhang, Keqin Hua

**Affiliations:** Department of Gynecology, Obstetrics and Gynecology Hospital of Fudan University, Shanghai, China

**Keywords:** cervical cancer, fertility-sparing surgery, radical trachelectomy, cerclage, intrauterine-cervical stent, pregnancy, delivery

## Abstract

**Background:**

Given the excellent prognosis of early-stage cervical cancer, fertility-sparing surgery has grown as a priority, significant alternative for radical hysterectomy in women being of reproductive age. We aimed to investigate the outcomes and subsequent pregnancies of early-stage cervical cancer patients who received radical trachelectomy. Moreover, there is a scarcity of literature directly comparing the impact of whether performing cervical cerclage concurrently with radical trachelectomy on patients’ reproductive outcomes.

**Methods:**

Women with IA1-IB2 cervical cancer who underwent fertility-sparing surgery at the Obstetrics and Gynecology Hospital of Fudan University were reviewed from January 2014 to May 2024.Radical trachelectomy in 70 women was performed by surgical team from the gynecologic oncologic center. Clinical characteristics, intraoperative, pathological results, oncologic, fertility and follow-up data of these patients were recorded and retrospectively analyzed. This study compared surgical and perinatal outcomes between patients who underwent cervical cerclage during radical trachelectomy (n=49) and those who did not receive the procedure simultaneously (n=21).

**Results:**

A total of 70 women (mean age: 31years) underwent radical trachelectomy (RT) of whom 68.6% were nulliparous. The FIGO stage distribution was IA1 (n=6), stage IA2 (n=7), stage IB1 (n=49), and stage IB2 (n=8). The operative duration was significantly longer in the cerclage group than in the control group (285.4±63.9 min vs 204.8±61.9 min; *p* < 0.001, 95% CI 47.51-113.48) with greater intraoperative blood loss (201.0 mL vs 170.1 mL, *p*=0.187, 95% CI -15.10-75.72). Overall, 36 women (51.4%) were seeking parenthood, and 26 succeeded (72.2%). There were 20 live births (76.9%), 12 women delivered in term (46.2%), 7 infants were born between 32 and 36+6 weeks, 1 between 28 and 31+6 weeks, all live birth. The mean neonatal birth weight was slightly lower in the cerclage group than in the control group (2625 g vs 2828.6 g; *p*=0.265, 95% CI -575.17 to 168.03). At the end of the follow-up period (median 68.7 months, range 34–153 months), one individual is currently 27+3 weeks pregnant, three patient had recurrence, and all women are alive and 20 children born to fertility-sparing surgery patients exhibited normal development.

**Conclusion:**

Radical trachelectomy provides excellent oncologic results with an outstanding fertility rate and obstetric outcome for patients with early cervical cancer. RT combined with intrauterine-cervical stent is a safe and effective fertility-sparing surgery but cervical cerclage is not recommended.

## Introduction

Cervical cancer (CC) is the most common gynecological malignancy in women and the leading cause of cancer death in some developing countries. Despite improvements in screening and prevention, CC remains a significant cause of morbidity and mortality (1). Studies have demonstrated an increasing occurrence rate of cervical cancer among women of reproductive age (2). Approximately 18.8%–28.1% of patients aged ≤45 years were subjected to fertility-sparing surgeries (e.g., cervical conization, simple or radical trachelectomy) (3). Among them, the proportion of patients under 40 years old undergoing such procedures increased from 17.8% to 28.1% (2006–2018) (4). In 2022, there were 151,000 new cases of cervical cancer in China, with an incidence rate of 13.8 per 100,000 people, showing a trend towards younger age groups (5). With the trend of delaying childbearing among Chinese women and the opening of the three-child policy (This new policy allows a couple to have three children), an increasing number of reproductive women with early-stage cervical cancer desire to retain their fertility without compromising overall survival.

Radical trachelectomy, as a fertility-preserving surgical option for patients with early-stage cervical cancer, is currently performed via two main approaches: open surgery and minimally invasive surgery (including laparoscopy and robot-assisted techniques). A large international multicenter retrospective study (2005 to 2017) demonstrated that the two approaches showed no significant differences in 4.5-year disease-free survival (99.2% vs. 99.0%) or overall survival, with low recurrence rates observed for both. Minimally invasive surgery has emerged as an alternative to open surgery due to its reduced surgical trauma (6). Existing literature reports a post-radical trachelectomy pregnancy rate of approximately 23.9%, with a live birth rate reaching 75.1% (7). Overall speaking, minimally invasive surgery is safe, effective, and particularly adapted for women who wish to preserve their fertility without compromising oncological and reproductive outcomes. However, due to its wider resection of the parametria and surgical resection range, post-operative cervical stenosis after RT was the most common reason for infertility after surgery (8). According to literature reports, the incidence of cervical canal adhesions can be as high as 33.3%(13/39) (9), which is not common in our study (2/70, 2.9%).

To maintain healthy menstruation and reduce infertility problems after radical trachelectomy, it is important to prevent postoperative cervical stenosis and cervical insufficiency. Until now, there have been few studies published fertility outcomes after radical trachelectomy and various surgical approaches, including vaginal RT (VRT), abdominal RT (ART), the robotic radical trachelectomy (RORT) and video laparoscopy radical trachelectomy (VLRT), yield different fertility outcomes (live delivery rate 8.33%-76.2%) (10–14).

Much of the current literature consists of case reports with limited sample sizes, lacking long-term follow-up data on oncologic outcomes and the health development of offspring. Recent reports, although documenting a larger number of surgeries, rarely mention fertility outcomes (15, 16). Furthermore, the procedure may compromise cervical competence, necessitating adjunctive techniques such as cervical stent placement and/or cervical cerclage to mitigate the risks of cervical insufficiency and cervical adhesion formation, which is rarely documented in detail in the literature (17). Here in our study, we found RT in our patients produces not only good oncological results but also promising pregnancy outcomes (pregnancy rate 72.2% and delivery rate 76.9% with normal development in offspring. We have summarized the key aspects of RT surgery for cervical cancer in our team, as follows:

1. Patients who are appropriately staged and aged (≤ IB2, ≤40 preferred);
2. Cervical cerclage may be performed either intraoperatively or during pregnancy, concurrent intraoperative cerclage is not routinely recommended;
3. Placement of an IUD and cervical stent during surgery to prevent cervical adhesion, highly recommended;
4. Rigorous postoperative follow-up and active encouragement of fertility at 6 months post-surgery when the intrauterine stent is removed.

Since initiating our radical trachelectomy program in 2009, we have performed over 100 procedures, observing merely three recurrences with zero mortality. Our team has previously established that fertility-sparing surgery (FSS) achieves favorable survival and prognostic outcomes for cervical cancer (18, 19). Furthermore, we recently published a dedicated analysis of oncologic management and outcomes in gravid cervical cancer patients (20). This present work reviews a decade of institutional data on oncologic and reproductive outcomes following radical trachelectomy. Crucially, our analysis demonstrates that pregnancy status does not adversely affect cervical cancer prognosis.

## Materials and methods

We retrospectively reviewed the medical records of patients diagnosed with cervical cancer who underwent radical trachelectomy in Shanghai tertiary medical center (Obstetrics and Gynecology Hospital of Fudan University) from January 2014 to May 2024.

Inclusion criteria were as follows: Patients who have strong desire to preserve fertility and no history of fertility impairment; aged 18 to 45 years; 2018 FIGO stage IA1-IB2; pathological types include adenocarcinoma, squamous cell carcinoma, or adenosquamous carcinoma; no evidence of upper endocervical involvement and a cranial extent of the tumor that is at least 1 cm away from the internal os; no lymph node spread or distant metastasis by preoperative imaging.

Exclusion criteria including: neuroendocrine carcinoma and gastric-type adenocarcinoma of the cervix; has received radiotherapy or neo-adjuvant therapy; tumors with internal os invasion or lymph node metastasis confirmed by intraoperative frozen section pathology; accompanied by any other kind of malignancy; absence of follow-up data.

**Figure 1:**
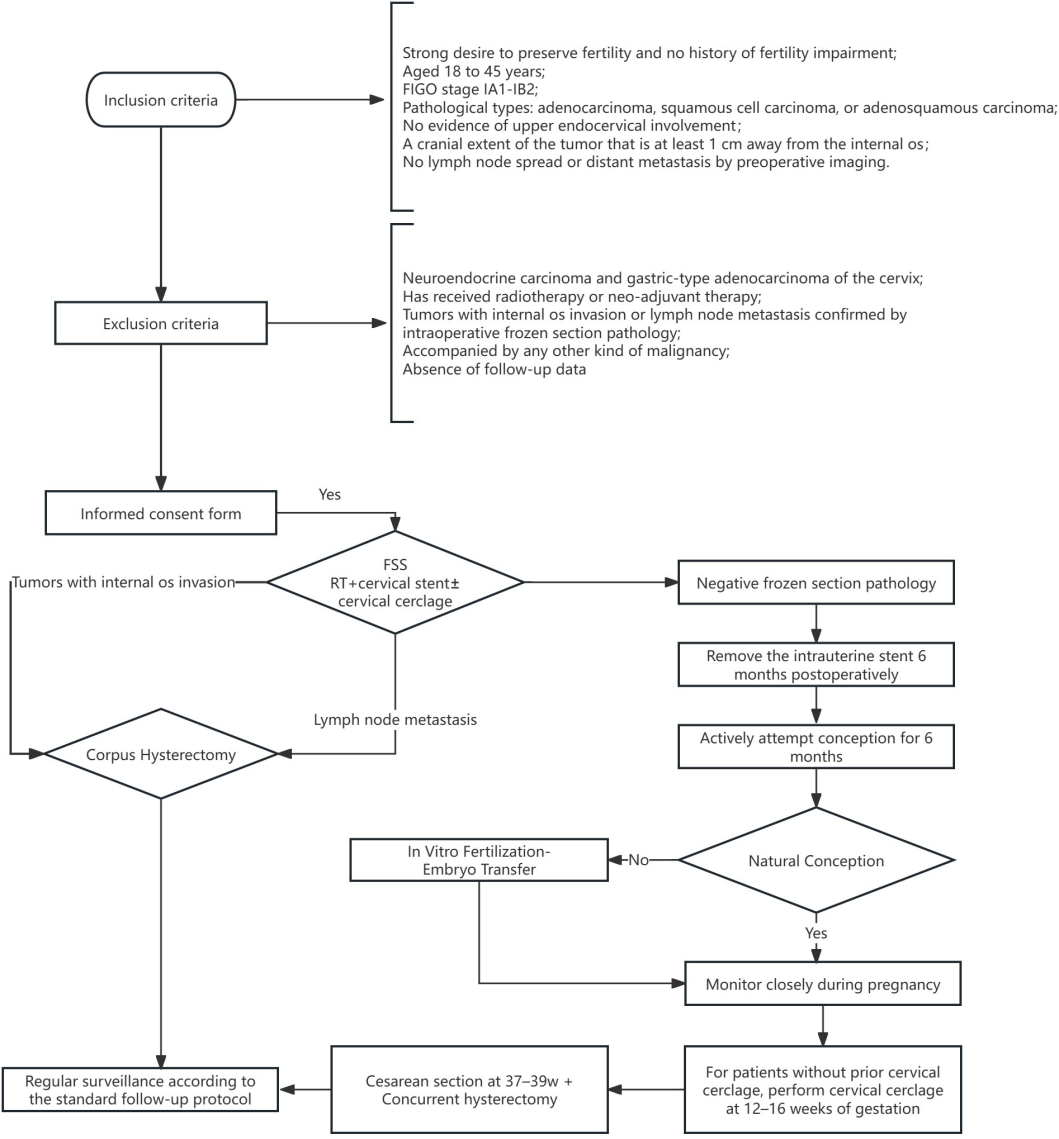
Therapeutic Protocol and Fertility Follow-up Algorithm for CC Patients Undergoing RT in this study. CC, cervical cancer; RT, radical trachelectomy; FIGO, International Federation of Gynecology and Obstetrics; FSS, fertility sparing surgery.

For the patients included in this study, we assessed the following variables: basic characteristics (age at diagnosis, BMI, FIGO stage, surgical approach and histology); perioperative outcomes (operation time, blood loss, hospital stay, and complication); fertility outcomes (pregnancy rate, natural conception rate and *in vitro* fertilization rate); obstetric outcomes (abortion, preterm birth, full-term birth, live birth and offspring health); oncologic prognosis (radiation/chemotherapy after CS and recurrences) at the end of the follow-up. Therapeutic protocol and fertility follow-up algorithm for cervical cancer patients undergoing RT in this study is shown in **Figure 1**. Two surgical procedures for cervical cancer were compared: Non-cerclage group and concurrent cervical cerclage group during RT.

### Preoperative assessment and follow-up

Two experienced gynecologic oncologists staged the patients and assessed parametrial involvement. Preoperative colposcopy was performed to exclude high-grade vaginal lesions or vaginal cancer. Preoperative imaging such as pelvic enhanced MRI were conducted to rule out pelvic metastasis. Follow-up data was obtained from the outpatient electronic medical record system, generally every three months for 2 years and every 6 months for 5 years after the surgery.

### Surgical procedure

#### Radical trachelectomy with pelvic lymph node dissection

Prior to the radical trachelectomy, a pelvic lymph node dissection is performed, and the excised common iliac lymph nodes are sent for rapid frozen section pathology examination. The radical trachelectomy proceeds only after pathological confirmation of tumor-free pelvic lymph nodes, while preserving the uterine artery’s upper branches. The parametrial tissue is cut 3cm from the uterus at the level of the uterosacral ligament and cardinal ligament. The cervix is transected at 30mm below the external cervical os, ligating and closing the cervical lesion, cutting the vaginal wall, and then dividing the cervix at the level of the internal cervical os. The excised cervix is sent for frozen section pathology examination through the vagina. If the cervical margins(>3-5mm), vaginal margins, and lymph nodes are all negative, the lower segment of the uterus and the posterior vaginal stump are further sutured.

#### Cervical stent insertion and cerclage

**Figure 2:**
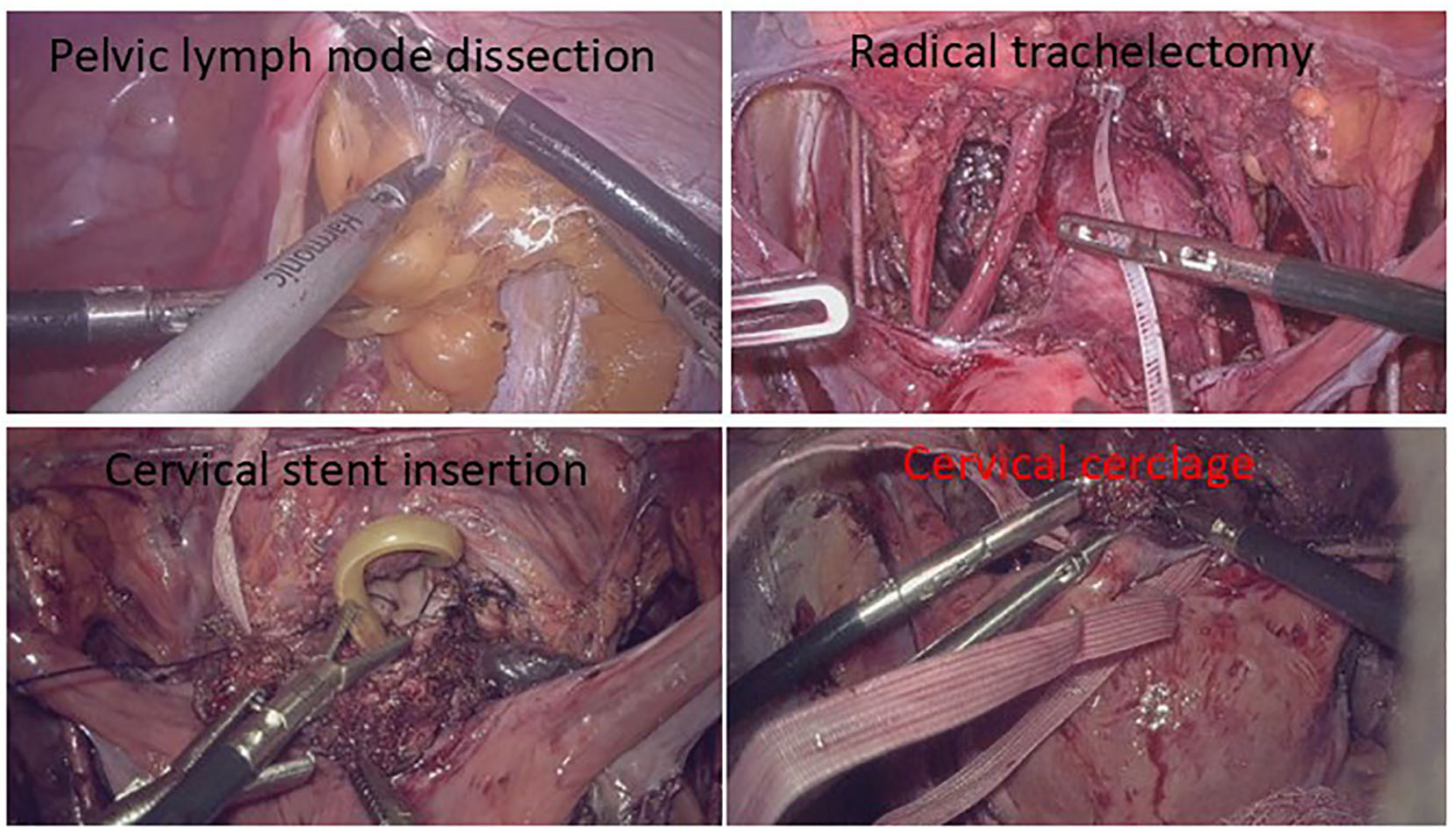
Surgical procedure flow. Pelvic lymphadenectomy is performed. Proceed with radical trachelectomy only upon negative lymph nodes. Implant homemade uterine-cervical stent into the uterine cavity and cervical os. Tighten the cerclage band and secure with a knot.

**Figure 3:**
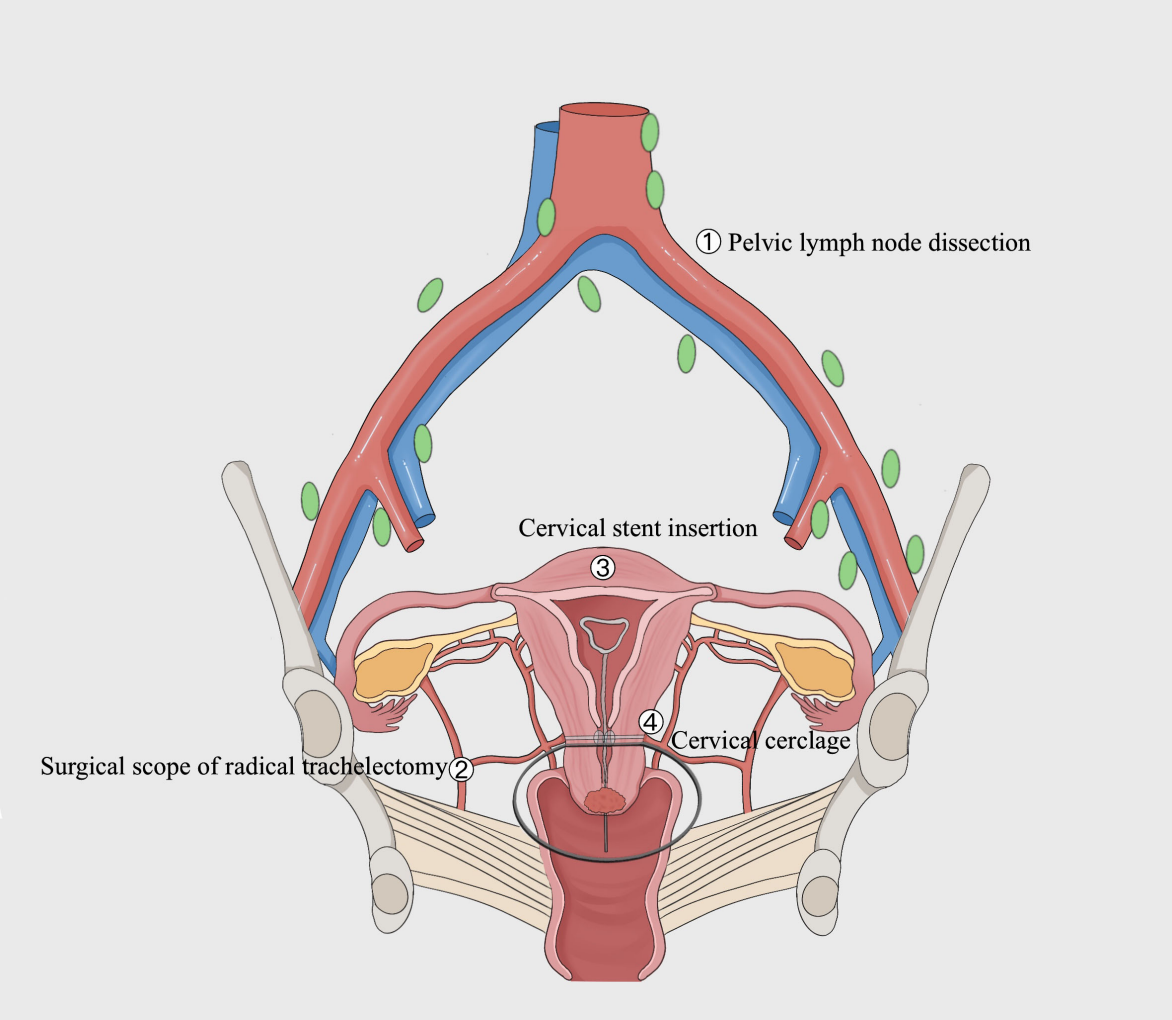
Schematic illustration of FSS in this study. As shown in the figure below, the surgical scope is marked by a black solid line. The surgery encompassed pelvic lymph node dissection and the excision of the cervix as well as 2-3cm of the vagina and adjacent para-uterine structures, while preserving the cervical tissue, specifically maintaining a distance of over 1cm from the internal os of the cervix. During the surgery, a cervical stent was applied, and cervical cerclage was performed simultaneously.

A polypropylene loop ligature is placed below the uterine artery at the internal cervical os, and an intrauterine device (IUD) is connected to a size 18 Foley catheter to create a homemade uterine-cervical stent, which is then implanted into the uterine cavity and cervical os. Tighten the cerclage band and secure with a knot. The peritoneal layer is sutured continuously with absorbable sutures. The detailed surgical procedure and schematic illustration of FSS in this study are illustrated in **Figure 2** and **Figure 3**.

### Statistical analysis

The t-test was used to compare continuous variables that followed a normal distribution and met the homogeneity of variance assumption, including age, BMI, operative time, blood loss, and neonatal birth weight, etc. For categorical variables (such as stage, histology, pregnancy rate, etc.), use the χ2 test or Fisher’s exact test, for continuous data that do not follow a normal distribution, use the Mann-Whitney U test. P-values <0.05 were considered to denote statistical significance.

## Results

### Baseline characteristics

The average follow-up time for these 70 patients was 68.7 months, with the longest follow-up time being 153 months. A total of 70 patients of reproductive age with stage IA1-IB2 cervical cancer underwent fertility-sparing surgery with a median follow-up of 5.7 years (range: 2.8-12.8 years). The median age of patients was 31.1 years, and 29.6% were primipara, with only 4 cases having received HPV vaccination (5.7%). Minimally invasive techniques (laparoscopic/robotic/gasless laparoscopic) constituted a majority of procedures (98.6%), contrasting with open abdominal surgery as the small minority approach (1.4%). The mean length of hospital stay was 10.6 days for the FSS patients, as shown in **Table 1**.

**Table 1:** Basic characteristics.

Patients	N=70(%)
Follow up months, (median, range)	68.7(34-153)
HPV vaccine	
Yes	4/70 (5.7%)
No	66/70 (94.3%)
Prior LEEP or CKC	
No	25/70 (35.2%)
LEEP	34/70 (47.9%)
CKC	11/70 (15.5%)
Surgical approach	
Laparoscopic	55/70(78.6%)
Robotic	12/70 (16.9%)
Gasless laparoscopic	2/70 (2.8%)
Abdom	1/70 (1.4%)
Post-operative hospital stay days, (median, range)	10.6(5-16)

CKC, cold knife conization; LEEP, loop electrosurgical excision procedure.

### Surgical outcomes

The mean ages of the non-cerclage group and cerclage group were 31.3 years and 30.6 years, respectively. No significant differences were observed between the cerclage and non-cerclage groups regarding age, BMI, FIGO stage, or pathological type (all p>0.05). Preoperative abortion rates and obstetric history also showed no intergroup differences. Intrauterine stent placement was performed concurrently in 83.7% of the cerclage group cases, significantly higher than the 52.4% rate in the non-cerclage group (p>0.05). As anticipated, the operative time in the cerclage group (285.4±63.9 min) was significantly longer than that in the non-cerclage group (204.8±61.9 min, p<0.001, 95% CI 47.51298,113.48022). Similarly, intraoperative blood loss was greater in the cerclage group (201.0ml *vs* 170.1ml, *p=*0.187, 95% CI -15.104,75.717). Notably, 2/49(4.1%)patients experienced complication in the cerclage group, whereas none were recorded in the non-cerclage group.

No differences were found between groups regarding lesion size (*p=*0.795), lymphovascular space invasion (LVSI) positivity rate (*p=*0.403), or depth of stromal invasion (*p=*0.723). The mean vaginal resection margin length was approximately 2 cm in both groups, with no significant differences at any measurement point (all p>0.05).

The number of common iliac lymph nodes harvested was approximately 1 bilaterally in both groups, showing no intergroup difference (left, *p=*0.401, right, *p=*0.320). The mean number of left pelvic lymph nodes retrieved was 6.9 in the cerclage group versus 8.1 in the non-cerclage group (*p=*0.142, 95% CI -2.977 to 0.437). For right pelvic lymph nodes, the counts were identical (7.7 nodes per group, *p=*0.935, 95% CI -2.151 to 2.335). We further compared the postoperative chemoradiotherapy status between the two groups, and the results revealed that postoperative chemotherapy rates were 2/21 (9.5%) in the cerclage group and 11/49 (22.4%) in the non-cerclage group, with no statistically significant difference (*p=*0.317). As shown in **Table 2**.

**Table 2:** Surgical data and complications.

Variables	Non-cerclage	Cerclage during RT	*p*	95% CI
Age (years)	31.3±6.3	30.6±4.7	0.577	-3.477,1.953
BMI (kg/m2)	22.5±2.8	20.5±2.4	0.122	-4.573,0.592
SCC antigen level	0.8(0.7,1.2)	0.8(0.6,1.1)	0.534	
Histological type			0.679	
SCC	15/21(71.4%)	38/49(77.6%)		
Adeno	4/21(19.0%)	9/49(18.4%)		
Adenosqmaous	2/21(9.5%)	2/49(4.1%)		
FIGO Stage, n (%)			0.133	
IA1	4/21(19.0%)	2/49(4.1%)		
IA2	3/21(14.3%)	4/49(8.2%)		
IB1	13/21(61.9%)	36/49(73.5%)		
IB2	1/21(4.8%)	7/49(14.3%)		
Pre-operative abortions	0(0,1.5)	1(0,2)	0.07	
Parous before FSS	6/21(28.6%)	16/49(32.7%)	0.736	
Nulliparous before FSS	15/21(71.4%)	33/49(67.3%)		
IUD+cervical stent	11/21(52.4%)	41/49(83.7%)	0.006	
Operative time min	204.8±61.9	285.4±63.9	<0.001	47.51298,113.48022
Intraoperative blood loss mL	170.1±59.3	201.0±96.5	0.187	-15.104,75.717
Intraoperative complications	0/21(0%)	2/49(4.1%)	NA	
Tumor size cm	0(0,2.5)	0(0,1.6)	0.795	
Lymph-vascular space invasion	4/21 (19.0%)	14/49(28.6%)	0.403	
Stromal invasion			0.723	
None	13/21(61.9%)	24/49(49.0%)		
Superficial	5/21(23.8%)	17/49(34.7%)		
Middle	1/21(4.8%)	4/49(8.2%)		
deep	2/21(9.5%)	4/49(8.2%)		
Histologic Subtype			0.985	
NA	15/21(71.4%)	34/49(69.4%)		
Keratinizing	2/21(9.5%)	5/49(10.2%)		
Non-keratinizing	4/21(19.0%)	10/49(20.4%)		
Length of vaginal cuff resection				
0 o’clock	2.2±0.8	2.0±0.8	0.319	-0.224,0.675
3 o’clock	2.2±0.6	2.3±0.7	0.552	-0.274,0.507
6 o’clock	2.3±0.7	2.7±0.8	0.073	-0.004,0.860
9 o’clock	2.1±0.6	2.3±0.7	0.204	-0.140,0.640
Number of common iliac lymph nodes				
left	1(1,2)	1(1,2)	0.401	
right	1(1,2.75)	1(1,2)	0.320	
Number of pelvic lymph nodes				
left	8.1±4.5	6.9±2.3	0.142	-2.977,0.437
right	7.7±3.7	7.7±4.1	0.935	-2.151,2.335
Postoperative chemotherapy	2/21(9.5%)	11/49(22.4%)	0.317	

Adeno, adenocarcinoma; FIGO, International Federation of Gynecology and Obstetrics; SCC, squamous cell carcinoma; FSS= fertility sparing surgery. NA, Not Available.

### Oncological and reproductive outcomes

Latest follow-up data revealed no recurrence in the non-cerclage group, whereas 3 cases of recurrence were observed in the concurrent intraoperative cerclage group, though the difference was not statistically significant (*p=*0.549). No mortality occurred in either group. The pregnancy attempt rates were comparable between groups (57.1% *vs*. 49.0%, *p*=0.531). The conception rate in the non-cerclage group was slightly higher (75.0% *vs*. 70.8%, *p*=0.793). Notably, the natural conception rate was higher in the non-cerclage group (77.8% *vs*. 52.9%, *p*=0.210), albeit without statistical significance.

No intergroup differences were observed in postoperative miscarriage frequency (*p*=0.788). The first-trimester miscarriage rate was 2/9 (22.2%) in the non-cerclage group versus 3/17 (17.6%) in the cerclage group. Mid-trimester intrauterine fetal demise and preterm birth rates were comparable (11.1% *vs*. 11.8% and 33.3% *vs*. 29.4%, respectively, *p=*0.252). Further comparison of live birth rates showed no significant difference (77.8% *vs*. 76.5%, *p=* 0.564). The term delivery rate was marginally higher in the concurrent cerclage group (8/17, 47.1% *vs*. 4/9, 44.4%), though statistically insignificant (*p*=0.608).

All cesarean sections and their post-operative courses were uneventful. No differences were noted in neonatal Apgar scores at 1 minute (median 9 in both groups, *p=*0.173), indicating vigorous newborn status. Offspring sex ratios and median gestational ages at delivery (37 weeks in both groups) were similar(both *p*>0.05). Interestingly, the mean birth weight was lower in the concurrent cerclage group (2625 g *vs*. 2828.6 g, *p*=0.265).

To further evaluate cervical cerclage’s gestational prolongation effect, patients were reclassified by cerclage status (regardless of timing: concurrent intraoperative or during pregnancy). Analysis revealed that the cerclage group had a longer mean gestational age (36.3 weeks *vs*. 33.4 weeks) and higher mean birth weight (2719.74 g *vs*. 2250 g, both *p*>0.05). These findings support routine recommendation of cervical cerclage after radical trachelectomy, though concurrent intraoperative cerclage is not routinely advised. Cerclage during pregnancy appears safe and avoids complications associated with intraoperative cerclage (e.g., tape erosion, abnormal uterine bleeding and foreign body reactions).

The incidence of antenatal complications—such as velamentous cord insertion and gestational diabetes mellitus—showed no significant difference between groups (*p*=0.613). Postpartum assessments revealed no evidence of tumor recurrence or metastasis in either group. A majority of patients opted for uterine preservation for future fertility (71.4% *vs* 61.5%, *p*=0.656).

No patients required radiotherapy or chemotherapy following cesarean delivery. Over 10 years of follow-up confirmed normal offspring development in both groups, with no congenital malformations or neurodevelopmental disorders observed. As shown in **Table 3**.

**Table 3:** Oncology follow-up and fertility data.

Variables	Non-cerclage	Cerclage during RT	*p*	95% CI
Recurrence	0/21(0%)	3/49(6.1%)	0.549	
Death	0/7(0%)	0/13(0%)	NA	
Attempting pregnancy	12/21(57.1%)	24/49(49.0%)	0.531	
Pregnancy rate	9/12(75.0%)	17/24(70.8%)	0.793	
Conception			0.210	
Natural conception	7/9(77.8%)	9/17(52.9%)		
IVF-ET	2/9(22.2%)	8/17(47.1%)		
Postoperative complications	1/21(4.8%)	3/49(6.1%)	0.728	
Number of miscarriages after FSS			0.788	
0	6/9(66.7%)	11/17(64.7%)		
1	2/9(22.2%)	4/17(23.5%)		
2	1/9(11.1%)	1/17(5.9%)		
3	0/9(0%)	1/17(5.9%)		
Fertility results			0.252	
Miscarriage ≤12th WOG	2/9(22.2%)	3/17(17.6%)		
Miscarriage >12th ≤28th WOG	1/9(11.1%)	2/17(11.8%)		
Preterm birth (>28 th<37 th WOG)	3/9(33.3%)	5/17(29.4%)		
Live birth	7/9(77.8%)	13/17(76.5%)	0.564	
Full-term delivery	4/9(44.4%)	8/17 (47.1%)	0.608	
Apgar 1min	9(9,9)	9(9,9)	0.173	
Gender of offspring			0.848	
Boy	3/7(42.9%)	5/13(38.5%)		
Girl	4/7(57.1%)	8/13(61.5%)		
Week of gestation	37(35.5,37.2)	37(35,37.35)	0.842	
Mean birth weight/g	2828.6±341.4	2625±393.9	0.265	-575.169,168.027
Complications during pregnancy	1/7(14.3%)	4/13(30.8%)	0.613	
Retaining the uterus after childbirth	5/7(71.4%)	8/13(61.5%)	0.656	
Radiation/chemotherapy after CS	0/7(0%)	0/13(0%)	NA	
Average age of offspring months, (median, range)	122(71,187)	132(62.5,190)	0.905	
Developmental abnormalities in offspring	0/7(0%)	0/13(0%)	NA	

IVF-ET, *in vitro* fertilization and embryo transfer; WOG, week of gestation; CS, cesarean section.

## Discussion

Nowadays, fertility-preservation treatment strategies for patients with early stage cervical cancer are continuously evolving, and less radical surgeries based on the ConCerv criteria are becoming more acceptable. Additional and ongoing evidence is helping determine the impact of conservative procedures on oncologic and obstetric outcomes in these patients (21).

The literature reports that the most common procedure performed was radical vaginal trachelectomy (40.7%) with the highest clinical pregnancy rate (67.5%) (21). However, vaginal trachelectomy also reported a higher recurrence rate (22). Beside, there was no significant difference in the overall survival, recurrence rate and death rate between the minimally invasive surgery (MIS) and abdominal surgery groups (23). A systematic review reported that laparoscopic and open radical trachelectomy demonstrate comparable efficacy in treating patients with early-stage cervical cancer, with no significant differences observed in survival rates, tumor recurrence rates, or mortality. Furthermore, the two surgical approaches showed no statistically significant differences in pregnancy-related outcomes, indicating that radical trachelectomy represents a viable fertility-sparing treatment option for early-stage cervical cancer patients (23). Salman L also reported that all surgical approaches including vaginal, abdominal, or minimally invasive surgery for radical trachelectomy seem to an equivalent oncologic outcome compared to radical hysterectomy (21). Our study revealed similar results. In our study, fertility-preserving surgeries were mainly performed using minimally invasive techniques such as laparoscopy (78.6%) and robotic surgery (16.9%), with only 1 case undergoing open surgery (1.4%). In this study, there was only three recurrences after radical trachelectomy, with no deaths reported, suggesting that RT and cervical cerclage itself may not increase tumor recurrence risk. The 5-year disease-free survival (DFS) rate and overall survival (OS) rate for patients undergoing fertility-preserving surgery were 95.7% and 100%, respectively, which were higher than those reported in the literature (5-year DFS 94%, OS 97%) (24). Long-term follow-up (>10 years) also demonstrated normal offspring development in both groups, supporting the safety of radical trachelectomy with cervical cerclage. This may be due to the early-stage patients included in this study, as patients with stage IB2 who had positive margins on frozen section or LVSI reported in the postoperative pathology were excluded from the analysis.

The reported average rate of cervical stenosis after radical trachelectomy was 10.5%. Among patients with abdominal, vaginal, laparoscopic and robotic radical trachelectomy, the incidences of cervical stenosis were 11.0%, 8.1%, 9.3% and 0%, respectively while the use of uterine cavity stents can prevent intrauterine adhesions(4.6% *VS* 12.7%, P<0.001) (25). Jaimin S Shah’s team indicated that cervical stenosis rates as high as 33.3% (13/39 cases) (9), some domestic scholars also pointed out that in the population undergoing radical trachelectomy with simultaneous cerclage compared to those without cerclage, there is a higher risk of postoperative cervical stenosis and adhesions, but the difference is not statistically significant (26). However, some studies have found that long-term follow-up data showed using cerclage tape does not increase the risk of cervical stenoses and the 5-year overall and recurrence-free survival rates were 100% and 97%, respectively (27), which is similar to the result in this study. Our data showed that only 2 cases of intrauterine adhesions postoperatively (2.9%), both occurring in the initial cases of our fertility-sparing surgery series, prior to technique standardization (without intrauterine stent placement). Crucially, cervical adhesions were rare in our cases regardless of cerclage status. This is likely attributable to our protocol of concurrent intraoperative stent insertion which is removed at 6 months post-surgery, allowing sufficient cervical healing time. We therefore strongly recommend our customized intrauterine-cervical stent which is made of 18F Foley catheter.

Due to limited evidence, no relevant guidelines have been established for clinical reference regarding whether cervical cerclage should be performed simultaneously during fertility-preserving surgery for cervical cancer. Statistical analysis based solely on the presence or absence of cervical cerclage (regardless of timing) demonstrated that the cerclage group exhibited significantly higher mean gestational age at delivery (36.3 weeks) compared to the non-cerclage group (33.4 weeks), along with greater neonatal birth weights (2719.74g *vs* 2250g), further confirming cerclage’s efficacy in prolonging pregnancy (28). However, the concurrent intraoperative cerclage subgroup paradoxically showed lower offspring weights than the non-cerclage group (2625g *vs* 2828.6g), potentially attributable to small sample size or confounding factors. Additionally, while live birth rates were comparable between groups (77.8% *vs* 76.5%), the non-cerclage group demonstrated higher natural conception rates (77.8% *vs* 52.9%), implying potential effects of intraoperative cerclage on fertility function (29).

In cases of cerclage placed in patients undergo radical trachelectomy, the procedure is typically performed via transabdominal or laparoscopic approach. Consequently, the cerclage cannot be removed vaginally. What’s more, it has been reported that once the prophylactic cerclage is removed, re-suturing may not be feasible (30). Therefore, the timing of cerclage placement needs to be individualized. Notably, as shown here in our study, concurrent intraoperative cerclage did not improve conception or live birth outcomes. Alarmingly, two mid-trimester intrauterine fetal demises due to preterm premature rupture of membranes (PPROM) occurred in the cerclage group. One patient suffered PPROM at 22.2 and 24.2 weeks respectively, requiring hysterotomy twice without successful cerclage removal. The uterine scars resulting from the two mid-trimester hysterotomy further delayed the opportunity for childbirth, causing profound psychological trauma in this patient. Although microbiological workup ruled out infection, a causal relationship between persistent mechanical irritation by the retained cerclage band and PPROM cannot be excluded. Additionally, one cerclage patient developed recurrent postcoital bleeding and cervical polyps impairing fertility attempts. Based on these findings, we recommend that concurrent intrauterine-cervical stent placement during fertility-sparing trachelectomy but simultaneous cerclage is not recommended. Antenatal cerclage should be considered when indicated, which demonstrates fewer sexual dysfunction issues and equivalent obstetric outcomes.

The factors influencing postoperative pregnancy in patients are numerous, such as anatomical issues, cervical canal adhesions, abnormal uterine bleeding, sexual dysfunction, and psychological issues like fear of tumor recurrence, fear of sexual activity, concerns about pregnancy affecting tumor prognosis, etc (31–33). According to literature reports, there is a significant variation in pregnancy rates after fertility-preserving surgery for cervical cancer. However, the results of this study show that although patients expressed a strong desire for fertility before surgery, most patients did not actively pursue fertility postoperatively, and a small number even gave up on having children. This study revealed that only 36 patients (51.4%) expressed a desire for fertility postoperatively regardless of prior childbearing status before FSS. These results are consistent with the current survey on fertility intentions of Chinese married women, which revealed that only 11.9% has the willingness to give birth, even with relaxation of the one-child policy (34). Our results supports the recommendation that a preoperative consultation carried out with a reproductive endocrinologist for all patients considering RT (9). As of the manuscript revision deadline, we have been recently notified of one patient currently at 27+3 weeks gestation with an uneventful pregnancy, and in contrast, another who recently confirmed pregnancy but underwent medication abortion at 7 weeks gestation due to no immediate childbearing plans. The fertility postponement phenomenon reflects structural tensions between temporal demands of career consolidation and biological reproductive constraints.

For cervical cancer patients undergoing fertility-sparing treatment, the literature recommends planned cesarean delivery between 37 and 39 weeks of gestation (35). This recommendation aligns with the median delivery gestational age of 37 weeks observed in our study. Besides, patients following radical trachelectomy in our hospital achieved significantly higher rates of pregnancy (72.2%) and live birth (55.6%) compared to literature-reported baselines (36.2% and 23.5%, respectively) (36). This outcome may be attributable to our minimally invasive surgical approach and larger cohort size. One similar study also indicated that robotic fertility-sparing radical trachelectomy in women with early stage cervical cancer can achieve high fertility rate, low rate of premature deliveries and an acceptable rate of recurrence (27).

Our institution has implemented a specialized multidisciplinary fertility preservation clinic that delivers structured patient education on cervical cancer management while providing personalized preconception counseling. For patients failing to conceive spontaneously within six months post-treatment, we initiate timely assisted reproductive technology (ART) referrals. This structured pathway aims to support patients’ pursuit of post-therapeutic fertility goals, with anticipated enrichment of longitudinal reproductive outcome data.

## Limitations

Our study has several notable limitations. Firstly, due to its retrospective nature, there are inherent risks such as selection bias, potential missing data, and reliance on existing records. This may affect the accuracy and comprehensiveness of the data analysis. Secondly, although the sample size of 70 is reasonable for this specialized surgical procedure, it may limit the ability to detect statistically significant differences in subgroup analyses or rare events. Additionally, being a single-center study, the results might reflect specific institutional practices and patient populations, thus potentially restricting the generalizability of the findings.

## Data Availability

The raw data supporting the conclusions of this article will be made available by the authors, without undue reservation.
