# Supplementary figures and images for: A long-term retrospective analysis of oncologic and fertility outcomes in cervical cancer patients undergoing radical trachelectomy
Source: Front Oncol. 2025 Sep 11;15:1591923. doi: 10.3389/fonc.2025.1591923 (PMC12460114; doi:10.3389/fonc.2025.1591923)

## Slide 1
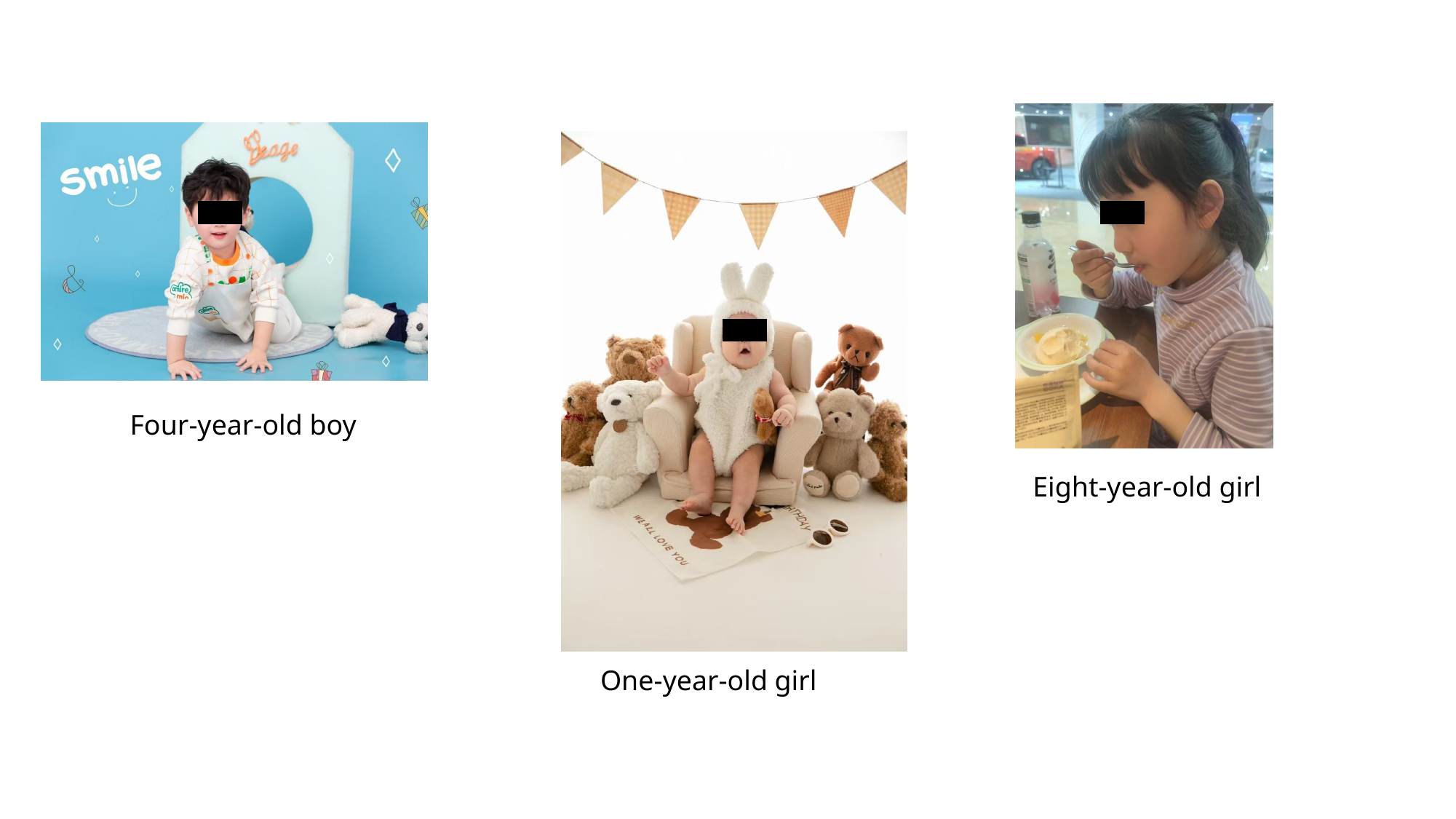

Four-year-old boy
Eight-year-old girl
One-year-old girl

Supplement: Supplementary file 1 [file Presentation1.pptx]
